# Clinical impact of routine response assessment after preoperative chemotherapy in patients with gastric cancer

**DOI:** 10.1093/bjsopen/zrad093

**Published:** 2023-09-20

**Authors:** Sander J M van Hootegem, Carlo A de Pasqual, Ben M Eyck, Bianca Mostert, Alexander Bradshaw, Alexander W Phillips, Sjoerd M Lagarde, Bas P L Wijnhoven

**Affiliations:** Department of Surgery, Erasmus MC University, Rotterdam, The Netherlands; Department of Surgery, Erasmus MC University, Rotterdam, The Netherlands; General and Upper GI Surgery Division, University Hospital of Verona, Verona, Italy; Department of Surgery, Erasmus MC University, Rotterdam, The Netherlands; Department of Medical Oncology, Erasmus MC Cancer Centre, Rotterdam, The Netherlands; Northern Centre for Cancer Care, Freeman Hospital, Newcastle-upon-Tyne, UK; Northern Oesophagogastric Unit, Royal Victoria Infirmary, Newcastle-Upon-Tyne, UK; School of Medical Education, Newcastle University, Newcastle-upon-Tyne, UK; Department of Surgery, Erasmus MC University, Rotterdam, The Netherlands; Department of Surgery, Erasmus MC University, Rotterdam, The Netherlands

## Introduction

Gastric cancer is one of the most common cancers worldwide and the third leading cause of cancer-related deaths^1^. As surgery is usually not advocated in patients with distant metastases, timely detection of disseminated disease can prevent futile surgery and may reduce complications and costs.

CT is routinely performed to exclude metastases; however, the sensitivity for the detection of (peritoneal) metastases is 22–33 per cent^2–4^. This explains the high rate (20–30 per cent) of occult metastases found with diagnostic laparoscopy in patients with locally advanced disease^5,6^.

Although routinely performed, the clinical impact of CT response assessment after preoperative chemotherapy is not well reported in patients with cancer of the stomach or oesophagogastric junction (OGJ). Incidental thromboembolic events are sometimes detected, requiring medical or other interventions and delay of surgery^7^. This study aimed to assess the clinical impact of routine CT for response assessment after preoperative chemotherapy in patients with cancer of the stomach or OGJ.

## Methods

Ethical approval was obtained from the local ethical committee (MEC-2019-0284). This study was performed according to the STROCCS criteria for cohort studies^8^. All patients with primary adenocarcinoma of the stomach or OGJ between January 2016 and December 2018 (with the bulk located in the stomach) and planned for perioperative chemotherapy plus surgery in two centres (Erasmus Medical Centre, Rotterdam, The Netherlands and the Northern Oesophagogastric Unit, Newcastle-upon-Tyne, UK) were included. Diagnostic laparoscopy with cytology of washings was indicated before commencing chemotherapy.

Endoscopic ultrasonography for clinical staging was done on indication (junctional cancers to assess infiltration of the oesophagus and distal cancers when there was doubt on infiltration of the duodenum). Fluor-18-deoxyglucose positron emission tomography was only performed in patients with advanced disease (T3-4 and/or cN1-3) or a tumour located at the OGJ.

The primary outcome was the proportion of patients in which CT findings led to a change in planned treatment. Secondary outcomes were the accuracy of CT in detecting interval metastases and the incidence of asymptomatic thromboembolic events on CT. To determine the accuracy of CT for the detection of metastases that preclude patients from surgical resection, the false-positive rate (FPR), false-negative rate (FNR), sensitivity, specificity, positive predictive value (PPV) and negative predictive value (NPV) were calculated on a per-patient level. For further details, see *Supplementary methods*.

## Results

**Fig. 1:**
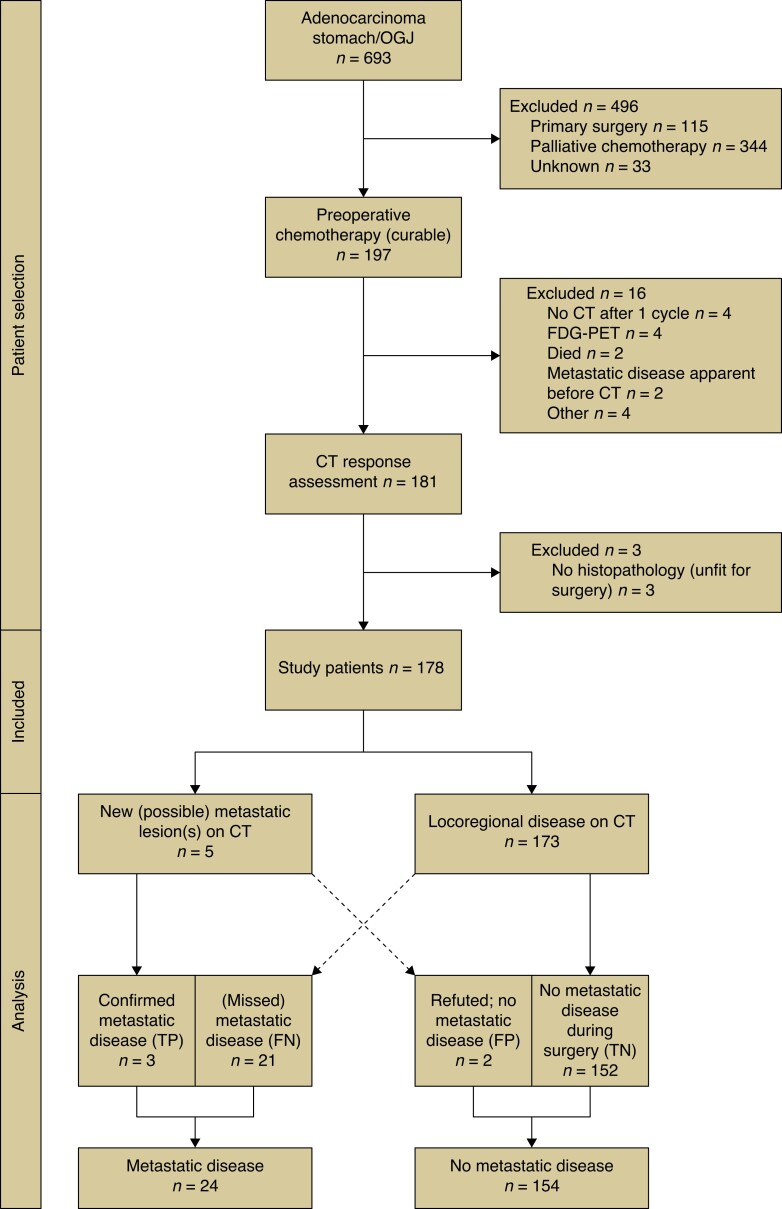
**Study flow chart** TP, true positives; FN, false negatives; FP, false positives; TN, true negatives; OGJ, oesophagogastric junction; FDG-PET, fluor-18-deoxyglucose positron emission tomography.

Of 693 patients with adenocarcinoma of the stomach or OGJ, 178 were included in the study after exclusions (*Fig. 1*, *Table 1*). Metastases or lesions suspicious for metastases were detected on CT in five of 178 patients. In two of five patients, metastatic disease was excluded with magnetic resonance imaging or (percutaneous) biopsy (false positive 2 of 178; 1.1 per cent) and in two of three patients metastatic disease was confirmed with histology (true positive 3 of 178; 1.7 per cent; details in *Table S1*). These three patients received non-surgical palliative treatment. No patients were diagnosed with locally irresectable disease on CT.

**Table 1:** Patient and treatment characteristics

	*n* = 178
Variables	*n* (%)
Age, years (median (i.q.r.))	66 (59–73)
**Sex**	
Male	135 (76)
Female	43 (24)
**Tumour location**	
OGJ	46 (26)
Proximal	45 (25)
Middle	25 (14)
Distal	41 (23)
Whole stomach	21 (12)
**Clinical T-stage**	
Tx	23 (13)
T1	1 (1)
T2	15 (8)
T3	71 (40)
T4a	65 (37)
T4b	3 (2)
**Clinical N-category**	
Nx	9 (5)
N0	53 (30)
N1	58 (33)
N2	36 (20)
N3	22 (12)
**Diagnostic laparoscopy**	
Yes	144 (81)
No	34 (19)
**Chemotherapy regimen (preoperative)**	
ECX/ECF*	154 (87)
Completed 3 cycles	123
Received >3 cycle(s)†	6
EOX/EOF	11 (6)
Completed 3 cycles	10
Received >3 cycles	1
FLOT‡	11 (6)
Completed 4 cycles	8
DCF	2 (1)
Completed 3 cycles	2

Values are *n* (%) unless otherwise indicated. Percentages may not total to 100 per cent because of rounding off. *Four patients switched from regimen: to EOX (*n* = 2), to cisplatin/paclitaxel (*n* = 1), or CAPOX (capecitabine and oxaliplatin; *n* = 1). †Five patients had multiple CTs after additional cycle(s) (2 CTs *n* = 4; 3 CTs *n* = 1). ‡One patient had an additional CT after an episode of neutropenic sepsis. i.q.r., interquartile range; OGJ, oesophagogastric junction; ECX/ECF, epirubicin, cisplatin, and either capecitabine (X) or fluorouracil (F); EOX/EOF, epirubicin, oxaliplatin, capecitabin (X)/fluorouracil (F); FLOT, fluorouracil, leucovorin, oxaliplatin, docetaxel; DCF, docetaxel, cisplatin, and fluorouracil.

Some 16 of 178 (9.0 per cent) patients had incidental thromboembolic events on CT, including pulmonary embolism in 13 and deep vein thrombosis in three. Eight patients had an inferior vena cava filter placed and planned treatment was continued. In four patients additional chemotherapy was given and surgery was postponed. Overall, planned treatment changed in 7 (3.9 per cent) of 178 patients who underwent CT response assessment.

Another 21 patients were diagnosed with metastases not detected by CT (false negative 21 of 178; 11.8 per cent, see *Table S2*). Some 154 of 172 patients had no signs of metastatic disease at the time of the operation (true negatives 154 of 178; 86.5 per cent). Overall, 24 (13.5 per cent) patients were diagnosed with metastatic disease after receiving preoperative chemotherapy (*Table S3*).

## Discussion

This study shows that a CT scan after preoperative chemotherapy had a limited yield in detecting interval metastases (1.7 per cent of patients) and more than 10 per cent of patients were additionally diagnosed with metastases during planned surgery.

In the FLOT4 trial, of 705 patients that received preoperative chemotherapy only two patients were diagnosed with metastatic disease by CT or MRI after preoperative chemotherapy^9^. Progressive disease or early death was seen in 17 patients, without specifying how this was diagnosed. Additionally, a total of 36 patients (5.2 per cent) did not undergo tumour resection despite being operated on, suggesting local irresectability or distant metastasis. In the MAGIC trial, the number of patients with non-resectional surgery after chemotherapy was 29 of 219 (13.2 per cent)^10^. These results support our findings that a routine CT scan cannot correctly identify patients with incurable disease after preoperative chemotherapy.

No major gastric cancer guidelines discuss the role of restaging after preoperative chemotherapy^11,12^. Accurate staging is of importance as surgical resection of gastric cancer with (limited) metastatic disease does not provide a survival benefit nor improvement in quality of life in most patients^13,14^. Additionally, unnecessary invasive surgery is associated with prolonged hospital stay and increased in-hospital morbidity and mortality rates^15^.

Metastases may be too small to detect with CT and become manifest in the following weeks before surgery. Nevertheless, other studies investigating the diagnostic performance of CT for clinical staging of peritoneal and abdominal metastases before treatment all show a low sensitivity and high specificity^2,4^. A diagnostic test with higher sensitivity would, therefore, be needed to reduce the substantial rate of false negatives found in this study, preventing non-resectional and futile surgery.

Diffusion weight magnetic resonance imaging (DW-MRI) may be able to detect peritoneal metastases more accurately with a sensitivity of 92 per cent in patients with gastrointestinal and ovarian cancer^16^. An alternative approach might be the application of radiomics. Using phenotype-reflecting features, derived from CT images of the primary tumour and nearby peritoneal region, improves the detection of occult metastases and could be easier to implement in clinical practice^17^. A pragmatic approach would be to perform a diagnostic laparoscopy with cytology of peritoneal fluid in all patients before a planned resection of the tumour as previously proposed by Thiels *et al.*^18^. Whilst still being able to take biopsies to confirm metastasis, it would reduce surgical trauma in some patients.

The rate of thromboembolism of 9.0 per cent in this study confirms that incidental thromboembolic events are common in this patient group^19^. Despite the development of several guidelines on thromboembolism in cancer patients, there is no clear support for routine administration of (prophylactic) anticoagulants in cancer patients undergoing neoadjuvant treatment^20^. Surgery early after a thromboembolic event poses risks of thromboembolic recurrence due to the need to temporarily hold anticoagulants and an increase in the morbidity rate associated with an embolic event, but postponing surgery could impair oncological outcomes. Consequently, there are many different approaches and the exact impact of these findings on treatment is therefore hard to determine.

## Supplementary Material

zrad093_Supplementary_Data

## Data Availability

The data sets used and analysed will be available upon reasonable request.
