# Supplementary material for: Clinical impact of routine response assessment after preoperative chemotherapy in patients with gastric cancer
Source: BJS Open. 2023 Sep 20;7(5):zrad093. doi: 10.1093/bjsopen/zrad093 (PMC10516457; doi:10.1093/bjsopen/zrad093)
Supplement: zrad093_Supplementary_Data [file zrad093_supplementary_data.docx]

**Clinical impact of routine response assessment after preoperative chemotherapy in patients with gastric cancer**

SJM van Hootegem^1^

CA de Pasqual^1,2^

BM Eyck^1^

B Mostert^3^

A Bradshaw^4^

AW Phillips^5,6^

SM Lagarde^1^

BPL Wijnhoven^1^

^1^Department of Surgery, Erasmus MC University, Rotterdam, The Netherlands

^2^ General and Upper GI Surgery Division, University Hospital of Verona, Verona, Italy

^3^Department of Medical Oncology, Erasmus MC Cancer Centre, Rotterdam, The Netherlands

^4^Northern Centre for Cancer Care, Freeman Hospital, Newcastle-upon-Tyne, UK

^5^Northern Oesophagogastric Unit, Royal Victoria Infirmary, Newcastle-Upon-Tyne, UK

^6^School of Medical Education, Newcastle University, Newcastle-upon-Tyne, UK

*Correspondence to:*

SJM van Hootegem

Department of Surgery, Erasmus MC, University Medical Center

P.O. Box 2040, 3000CA Rotterdam, The Netherlands

[s.vanhootegem@erasmusmc.nl](mailto:s.vanhootegem@erasmusmc.nl) telephone: +31642529735; fax number: 010-7032396

*Type of study:* short report *Sources of funding:* none

*Availability of data:* The datasets used and analysed will be available upon reasonable request.

**Supplementary Materials - Index**

| **Supplementary Methods** |  |
| --- | --- |
| Patients | *page 3* |
| Procedures | *page 3* |
| CT scan | *page 3* |
| Statistical analysis | *page 4* |
| Scanning details | *page 4* |
| **Supplementary Figures and Tables** |  |
| True positives | *page 5* |
| False negatives | *page 6* |
| Locations of metastases | *page 7* |
| **References** | *page 8-10* |
|  |  |

**Supplementary Methods**

*Patients*

All patients with adenocarcinoma of the stomach or OGJ who were discussed at multidisciplinary tumour boards (MDT) between January 2016 and December 2018 for upper gastrointestinal malignancy (from the Erasmus Medical Centre, Rotterdam, the Netherlands and the Northern Oesophagogastric Unit, Newcastle-upon-Tyne, UK) were retrospectively reviewed.

Patients were included if they had primary adenocarcinoma of the stomach or OGJ (with the bulk located in the cardia), were eligible for curative treatment (cT1N1-3M0 or cT2-4N0-3M0 with negative cytology of abdominal washings when DLS was performed) and were planned for perioperative chemotherapy plus surgery. At least 50% of the planned preoperative chemotherapy regimen had to be completed.

Exclusion criteria were no CT response assessment after preoperative chemotherapy done and the unavailability of histopathological results.

*Procedures*

The extent of disease was staged according to the 8^th^ edition of the Union for International Cancer Control TNM classification.^26^

Patients were generally treated according to the MAGIC regimen, consisting of three preoperative and three postoperative three-weekly cycles of epirubicin, cisplatin and either capecitabine or fluorouracil (ECC/ECF) (Table 1).^13^ Patients with poor renal and/or cardiac function were commonly administered oxaliplatin instead of cisplatin (EOX/EOF). The FLOT-regimen, consisting of four preoperative and four postoperative two-weekly cycles of fluorouracil, leucovorin, oxaliplatin and docetaxel was also allowed.^12^

After (or during) the last preoperative cycle a response assessment with CT was performed, and findings were discussed at the MDT. If deemed fit for surgical treatment and CT showed no signs of metastasis, patients proceeded to surgery, either surgical resection or re-staging laparoscopy.

Surgical resection was preferably performed 4-6 weeks after completion of chemotherapy and consisted of either (sub)total gastrectomy or oesophagectomy depending on tumour location and preference of the surgeon. Irrespective of the surgical technique, a D1+ or D2 lymphadenectomy was performed in all patients.

*CT scan*

As both institutions are tertiary referral hospitals, and some patients thus underwent CT-scans in another hospital, there was some variability in scanning protocols. Patients at both centres were scanned with multislice scanners from a single vendor (Siemens Healthineers) after fasting for four to six hours and being instructed to drink water to achieve gastric distention. Contrast-enhanced scans were performed in the portal venous phase (65-80s) after administration of weight-adjusted intravenous iodinated contrast. Appendix 1 shows descriptions of institutional scanning protocols. When the CT scan was done in the referring hospital, they were reviewed by radiologists from the MDT and if deemed of adequate quality, the CT was not repeated.

CT interpretations rendered as part of clinical care were extracted from MDT reports where an experienced gastrointestinal radiologist discussed all findings with MDT members after a second review of the original images and reports. In addition to a radiologist, members of the MDT included at least an upper GI surgeon, medical oncologist, radiation oncologist and gastroenterologist.

*Statistical analysis*

Categorical variables were reported as frequencies and percentages with 95% confidence intervals and distribution of continuous variables as medians with interquartile ranges (when non-parametric). To determine the accuracy of CT for the detection of metastases that preclude patients from surgical resection, the false-positive rate (FPR), false-negative rate (FNR), sensitivity, specificity, positive predictive value (PPV) and negative predictive value (NPV) were calculated and expressed as percentages with 95% confidence intervals (CI) on a per-patient level. Given the sample sizes (small and mid-range), 95% CIs were calculated according to the Wilson score procedure, without a correction for continuity.^27^ Metastases (according to TNM-staging criteria^26^) were defined as unequivocal new lesions or progression of lesions on the CT after chemotherapy as compared to the CT used for clinical staging, or highly suspicious lesions that needed additional investigations for characterization. Histopathology of a lesion from tissue(s) obtained with a percutaneous biopsy or during surgery was used as the reference standard. When multiple CT scans were performed after chemotherapy, the one closest to the date of surgery was used. Statistical analyses were performed using SPSS (Version 25.0. Armonk, NY: IBM Corp.).

*Scanning details*

The CT scanners used for study patients in the Erasmus MC were all 128- up to 192-multislice single source or dual source systems from a single vendor (Siemens Healthineers). All patients were fasted 4 hours before scanning. Contrast-enhanced scans were obtained using a dedicated gastric cancer/oncology protocol encompassing thorax and upper abdomen. Scans were performed in porto-venous phase 65-80s with weight adjusted (0.4-0.5 GI2/Kg) intravenous iodinated contrast (Iodixanol 320, GE Healthcare) and water distension of the stomach with patients in supine position. For reporting, standard soft tissue thick (3.0 mm axial, coronal and sagittal) and thin (1.0 mm axial), together with Hi-resolution lung (1.0 mm) images were reconstructed. Upon request, the protocol was extended to include an arterial upper abdomen scan and/or include the pelvis in the venous scan. Scans were performed with automated kV and mA modulation to optimize radiation dose to body size and habitus and image contrast to iodinated soft tissue scans.

All scans at the Northern Oesophagogastric Unitwere performed on Siemens multislice scanners (128/384 slice Force) or (128 slice Edge) using a single energy source. Patients were fasted for 6 hours and instructed to drink water prep (500mL over 30minutes) prior to the scan. Contrast enhanced scans were performed in the portal venous phase (65s) using Omnipaque 350 (2mL/kg at 3mL/s) (GE Healthcare) following the administration of buscopan 20mg IV. Images were obtained from the base of skull to the pubic symphysis. Automatic kV and mAs dose modulation (“Care kV” and “Caredose”) was used to optimise dose and image quality. Soft tissue reformats (1mm thick) in the axial plane were produced using “Admire 4” iterative reconstruction and a Br36 Kernal. Images were sent to PACS (Carestream) and reviewed in multiple planes using PACS intrinsic capabilities.

**Supplementary Figures and Tables**

| **Supplementary Table 1** True positives; patients with interval metastases detected by CT | | | | | | | | | |
| --- | --- | --- | --- | --- | --- | --- | --- | --- | --- |
| **Patients** | | *Age* | *Gender* | *Tumour histology** | *Tumour location* | *cTNM stage* | *Chemotherapy (cycles completed)* | *Metastatic location(s)* | *Additional investigation(s)* |
| 1. |  | 78 | Male | Diffuse | Proximal | T3N2M0 | ECX (3) | Non-regional nodes; para-aortic lymph node (left renal vein) | CT-guided biopsy: positive for metastasis |
| 2. |  | 61 | Male | Diffuse | Middle | T4aN3M0 | ECX (3) | Non-regional nodes; peripancreatic | No further confirmatory investigation(s) |
| 3. |  | 62 | Male | Intestinal | Body (linitis) | T4aN1M0 | ECX (3) | Hepatic lesion (1cm) | CT-guided biopsy: positive for metastasis |
| *ECX epirubicin, cisplatinum, capecitabine*  ** According to Lauren classification.* | | | | | | | | | |

| **Supplementary Table 2** False negatives; patients with interval metastases not detected by CT | | | | | | |  |  |  |
| --- | --- | --- | --- | --- | --- | --- | --- | --- | --- |
|  |  |  |  |  |  |  |  |  |  |
| **Patients** |  | *Age* | *Gender* | *Tumour histology** | *Tumour location* | *cTNM stage* | *Chemotherapy (cycles completed)* | *Metastatic location(s)* | *Tissue collected during/with* |
| 1. |  | 66 | Female | Diffuse | Middle | T3N1M0 | ECX (3) | Peritoneal | Surgery |
| 2. |  | 69 | Male | Diffuse | Body (linitis) | T2N0M0 | FLOT (4) | Peritoneal | DLS |
| 3. |  | 74 | Male | Diffuse | Proximal | T3N0M0 | ECX (2) | Peritoneal | Surgery |
| 4. |  | 61 | Male | Intestinal | Proximal | T3N1M0 | ECX (3) | Peritoneal + hepatic | Surgery |
| 5. |  | 44 | Female | Diffuse | Proximal | T3N0M0 | ECX (3) | Omental foci | Surgery |
| 6. |  | 76 | Female | Intestinal | Proximal | T2N1M0 | ECX (2) | Peritoneal, hepatic, omental and pelvic | CT-guided biopsy (after clinical symptoms) |
| 7. |  | 61 | Female | Intestinal | Distal | TxN1M0 | ECX (3) | Non-regional nodes; dorsal of pancreatic head | Surgery |
| 8. |  | 74 | Male | - | Distal | T3N0M0 | ECX (3) | Peritoneal | DLS |
| 9. |  | 65 | Male | Intestinal | Proximal | T4N3Mx | EOF (6) | Non-regional nodes; on retroperitoneum and near renal artery | Surgery |
| 10. |  | 60 | Male | - | OGJ | T3N0M0 | DCF (3) | Non-regional nodes; dorsal to celiac trunk | Surgery |
| 11. |  | 65 | Male | - | Distal | T4bN2M0 | ECX (2) + Cisplatin/Paclitaxel (6) | Non-regional nodes; near the ligament of Treitz and in the falciform ligament (+ local invasion into the pancreas and mesocolon) | Surgery |
| 12. |  | 67 | Female | Diffuse | Distal | T4N3M0 | ECX (3) | Peritoneal | Surgery |
| 13. |  | 61 | Male | - | Proximal | TxN0M0 | ECX (3) | Peritoneal (Sister Mary Joseph nodule) | Percutaneous biopsy (after clinical symptoms) |
| 14. |  | 62 | Male | Intestinal | Proximal | T3N1M0 | ECX (3) | Peritoneal | DLS (after clinical symptoms) |
| 15. |  | 47 | Male | Mixed | OGJ | TxN1M0 | DCF (3) | Omental (+ non-intraperitoneal para-oesophageal node) | Surgery |
| 16. |  | 54 | Male | Intestinal | OGJ | T4N1M0 | ECX (3) | Hepatic | Surgery |
| 17. |  | 62 | Male | Diffuse | Middle | T4N0M0 | ECX (3) | Peritoneal | DLS |
| 18. |  | 52 | Male | Intestinal | OGJ | T4N2M0 | ECX (3) | Peritoneal | DLS |
| 19. |  | 86 | Male | Intestinal | Middle | T3N2M0 | ECX (3) | Peritoneal | DLS |
| 20. |  | 52 | Male | Diffuse | Body (linitis) | T4N1M0 | ECX (3) | Separate serosal deposits | Surgery |
| 21. |  | 70 | Male | Diffuse | Body (linitis) | T4N1M0 | FLOT (2) | Hepatic | Surgery |
| *DLS diagnostic laparoscopy; OGJ oesophagogastric junction ECX/ECF epirubicin, cisplatin and either capecitabine (X) or fluorouracil (F); EOX/EOF epirubicin, oxaliplatin, capecitabin (X)/fluiorouracil (F); FLOT fluorouracil, leucovorin, oxaliplatin, docetaxel ; DCF Docetaxel, cisplatin and fluorouracil* | | | | | | | | | |
| **According to Lauren Classification.* | | |  |  |  |  |  |  |  |

| **Supplementary Table 3** Locations of metastases | | | |  |
| --- | --- | --- | --- | --- |
| **Location** |  |  |  | *n* |
| ***Detected by CT (TP)*** |  |  |  |  |
| Lymph nodes (non-regional) | |  |  | 2 |
| Hepatic (solitary) | |  |  | 1 |
|  |  |  |  |  |
| ***Not detected by CT (FN)*** | |  |  |  |
| Peritoneal |  |  |  | 12 |
| Lymph nodes (non-regional) | |  |  | 4 |
| Peritoneal and hepatic | |  |  | 2 |
| Hepatic | |  |  | 2 |
| Separate serosal deposits | |  |  | 1 |
|  | |  |  |  |
| ***Total*** | |  |  | 24 |
| *CT Computed tomography; TP true positives; FN false negatives* | | | | |
|  | | | | |

**References**

1. Bray F, Ferlay J, Soerjomataram I, Siegel RL, Torre LA, Jemal A. Global cancer statistics 2018: GLOBOCAN estimates of incidence and mortality worldwide for 36 cancers in 185 countries. CA Cancer J Clin. 2018;68:394-424.

2. Burbidge S, Mahady K, Naik K. The role of CT and staging laparoscopy in the staging of gastric cancer. Clin Radiol. 2013;68:251-5.

3. Smyth E, Schoder H, Strong VE, Capanu M, Kelsen DP, Coit DG, et al. A prospective evaluation of the utility of 2-deoxy-2-[(18) F]fluoro-D-glucose positron emission tomography and computed tomography in staging locally advanced gastric cancer. Cancer. 2012;118:5481-8.

4. Kim SJ, Kim HH, Kim YH, Hwang SH, Lee HS, Park DJ, et al. Peritoneal metastasis: detection with 16- or 64-detector row CT in patients undergoing surgery for gastric cancer. Radiology. 2009;253:407-15.

5. Feussner H, Omote K, Fink U, Walker SJ, Siewert JR. Pretherapeutic laparoscopic staging in advanced gastric carcinoma. Endoscopy. 1999;31:342-7.

6. Lowy AM, Mansfield PF, Leach SD, Ajani J. Laparoscopic staging for gastric cancer. Surgery. 1996;119:611-4.

7. Khanna A, Reece-Smith AM, Cunnell M, Madhusudan S, Thomas A, Bowrey DJ, et al. Venous thromboembolism in patients receiving perioperative chemotherapy for esophagogastric cancer. Dis Esophagus. 2014;27:242-7.

8. Mathew G, Agha R, Group S. STROCSS 2021: Strengthening the reporting of cohort, cross-sectional and case-control studies in surgery. Int J Surg. 2021;96:106165.

9. Al-Batran SE, Homann N, Pauligk C, Goetze TO, Meiler J, Kasper S, et al. Perioperative chemotherapy with fluorouracil plus leucovorin, oxaliplatin, and docetaxel versus fluorouracil or capecitabine plus cisplatin and epirubicin for locally advanced, resectable gastric or gastro-oesophageal junction adenocarcinoma (FLOT4): a randomised, phase 2/3 trial. Lancet. 2019;393:1948-57.

10. Cunningham D, Allum WH, Stenning SP, Thompson JN, Van de Velde CJ, Nicolson M, et al. Perioperative chemotherapy versus surgery alone for resectable gastroesophageal cancer. N Engl J Med. 2006;355:11-20.

11. Ajani JA, D'Amico TA, Almhanna K, Bentrem DJ, Chao J, Das P, et al. Gastric Cancer, Version 3.2016, NCCN Clinical Practice Guidelines in Oncology. J Natl Compr Canc Netw. 2016;14:1286-312.

12. Smyth EC, Verheij M, Allum W, Cunningham D, Cervantes A, Arnold D, et al. Gastric cancer: ESMO Clinical Practice Guidelines for diagnosis, treatment and follow-up. Ann Oncol. 2016;27:v38-v49.

13. Ouchi K, Sugawara T, Ono H, Fujiya T, Kamiyama Y, Kakugawa Y, et al. Therapeutic significance of palliative operations for gastric cancer for survival and quality of life. J Surg Oncol. 1998;69:41-4.

14. Fujitani K, Yang HK, Mizusawa J, Kim YW, Terashima M, Han SU, et al. Gastrectomy plus chemotherapy versus chemotherapy alone for advanced gastric cancer with a single non-curable factor (REGATTA): a phase 3, randomised controlled trial. Lancet Oncol. 2016;17:309-18.

15. Karanicolas PJ, Elkin EB, Jacks LM, Atoria CL, Strong VE, Brennan MF, et al. Staging laparoscopy in the management of gastric cancer: a population-based analysis. J Am Coll Surg. 2011;213:644-51, 51 e1.

16. van 't Sant I, van Eden WJ, Engbersen MP, Kok NFM, Woensdregt K, Lambregts DMJ, et al. Diffusion-weighted MRI assessment of the peritoneal cancer index before cytoreductive surgery. Br J Surg. 2019;106:491-8.

17. Liu S, He J, Liu S, Ji C, Guan W, Chen L, et al. Radiomics analysis using contrast-enhanced CT for preoperative prediction of occult peritoneal metastasis in advanced gastric cancer. Eur Radiol. 2020;30:239-46.

18. Thiels CA, Ikoma N, Fournier K, Das P, Blum M, Estrella JS, et al. Repeat staging laparoscopy for gastric cancer after preoperative therapy. J Surg Oncol. 2018;118:61-7.

19. Aonuma AO, Nakamura M, Sakamaki K, Murai T, Matsuda C, Itaya K, et al. Incidence of cancer-associated thromboembolism in Japanese gastric and colorectal cancer patients receiving chemotherapy: a single-institutional retrospective cohort analysis (Sapporo CAT study). BMJ Open. 2019;9:e028563.

20. Mandala M, Falanga A, Roila F, Group EGW. Management of venous thromboembolism (VTE) in cancer patients: ESMO Clinical Practice Guidelines. Ann Oncol. 2011;22 Suppl 6:vi85-92.
